# Pulmonary function with expiratory resistive loading in healthy volunteers

**DOI:** 10.1371/journal.pone.0252916

**Published:** 2021-06-11

**Authors:** Jyotika Erram, Monica Bari, Antoinette Domingo, Daniel T. Cannon

**Affiliations:** School of Exercise & Nutritional Sciences, San Diego State University, San Diego, California, United States of America; Mayo Clinic College of Medicine, UNITED STATES

## Abstract

Expiratory flow limitation is a key characteristic in obstructive pulmonary diseases. To study abnormal lung mechanics isolated from heterogeneities of obstructive disease, we measured pulmonary function in healthy adults with expiratory loading. Thirty-seven volunteers (25±5 yr) completed spirometry and body plethysmography under control and threshold expiratory loading of 7, 11 cmH_2_O, and a subset at 20 cmH_2_O (n = 11). We analyzed the shape of the flow-volume relationship with rectangular area ratio (RAR; Ma *et al*., *Respir Med* 2010). Airway resistance was increased (*p*<0.0001) with 7 and 11 cmH_2_O loading vs control (9.20±1.02 and 11.76±1.68 vs. 2.53± 0.80 cmH_2_O/L/s). RAR was reduced (*p* = 0.0319) in loading vs control (0.45±0.07 and 0.47±0.09L vs. 0.48±0.08). FEV_1_ was reduced (*p*<0.0001) in loading vs control (3.24±0.81 and 3.23±0.80 vs. 4.04±1.05 L). FVC was reduced (*p*<0.0001) in loading vs control (4.11±1.01 and 4.14±1.03 vs. 5.03±1.34 L). Peak expiratory flow (PEF) was reduced (p<0.0001) in loading vs control (6.03±1.67 and 6.02±1.84 vs. 8.50±2.81 L/s). FEV_1_/FVC (*p*<0.0068) was not clinically significant and FRC (p = 0.4) was not different in loading vs control. Supra-physiologic loading at 20 cmH_2_O did not result in further limitation. Expiratory loading reduced FEV_1_, FVC, PEF, but there were no clinically meaningful differences in FEV_1_/FVC, FRC, or RAR. Imposed expiratory loading likely leads to high airway pressures that resist dynamic airway compression. Thus, a concave expiratory flow-volume relationship was consistently absent–a key limitation for model comparison with pulmonary function in COPD. Threshold loading may be a useful strategy to increase work of breathing or induce dynamic hyperinflation.

## Introduction

Chronic obstructive pulmonary disease (COPD) and asthma are broadly characterized by airway narrowing, high airway resistance, expiratory flow limitation, and exercise intolerance. Expiratory flow limitation in obstructive disease often leads to abnormal lung mechanics such as static and dynamic hyperinflation—powerful drivers of dyspnea [1–3]. However, obstructive diseases are exceedingly heterogeneous. For example, patients with COPD suffer from a variety of abnormalities in skeletal muscle [4–6], cardiovascular dysfunction [7], physical inactivity [8, 9], and high systemic inflammatory burden [10]. Isolating the effects of expiratory flow limitation on lung mechanics alone is challenging for these and other reasons.

For restrictive diseases such as pulmonary fibrosis and interstitial lung disease, chest wall strapping is a simple and useful model for understanding lung mechanics by reducing lung volumes and increasing chest wall stiffness [11]. Such a simple solution for obstructive diseases is elusive. Expiratory resistive loading is effective for increasing the work of breathing [12], dyspnea, exercise intolerance [13–15] and is useful with or without metronome-paced tachypnea for inducing dynamic hyperinflation in healthy volunteers [16]. This is true for both reduced aperture or Starling resistors [16, 17]. Reducing the aperture during expiration is especially effective for reproducing the characteristic flow-volume deficits in upper airway obstruction [18, 19], however upper airway obstruction is very different in presentation of the flow-volume loop than small airway disease such as COPD.

Imposing adjustable external expiratory resistance with a threshold device [15] may provide a non-invasive method to simulate the effects of obstruction and to serve as a model for studying abnormal lung mechanics at rest and during exercise. This could provide supplemental experimental model options to that of either reduced aperture or Starling resistors for expiratory loading [14, 16, 17]. A comprehensive pulmonary function assessment is not available in the literature for examining healthy people with imposed threshold pressure expiratory loading. Thus, we aimed to measure the effects of expiratory loading on pulmonary function in young, healthy volunteers. We hypothesized that external expiratory loading would produce dose-response impairment in the expiratory segment of the flow-volume envelope, similar to what is present in those with obstructive diseases.

## Materials and methods

### Study participants

Forty-three healthy adults were recruited to participate (25 ± 5 yrs, 171.8 ± 10.2 cm, 72.2 ± 13.3 kg, 24 men, 19 women). Volunteers were included on the basis of age, 18 years and older, and were screened for cardiopulmonary disease using the physical activity readiness questionnaire (PAR-Q). Exclusion criteria included abnormal spirometry defined as an FEV_1_/FVC < 0.70. Written informed consent was obtained and the study protocol was approved by the Institutional Review Board of San Diego State University.

### Spirometry and plethysmography

Participants completed spirometry and whole-body plethysmography under control conditions and with imposed expiratory threshold loading at 7 and 11 cmH_2_O (n = 43). A subgroup performed an additional spirometry condition with a loading at 20 cmH_2_O (n = 15). The expiratory load was produced with a threshold expiratory training device (Threshold PEP, Respironics, Pittsburgh, PA) installed between the pulmonary filter and pneumotachometer. The threshold device relies on a flow-independent one-way valve that provides constant pressure that is adjustable based on the valve spring tension. The manufacturer’s accuracy and reproducibility are ± 1.0 and ± 0.5 cmH_2_O, respectively. Both the spirometer and the plethysmograph were calibrated with a 3L syringe according to manufacturer’s instructions. Spirometry (TrueOne, Parvo Medics, Sandy, UT) and plethysmography were measured using commercial PFT systems (Vmax, CareFusion, Yorba Linda, CA) according to ATS/ERS standards. Pulmonary function was evaluated by forced expiratory volume in the first second (FEV_1_), forced vital capacity (FVC), ratio of the forced expiratory volume in the first second to forced vital capacity (FEV_1_/FVC), and peak expiratory flow rate (PEFR) produced by spirometry and airway resistance (R_aw_)measured by plethysmography.

During spirometry testing, participants were seated upright, holding the pneumotachometer to their mouth and wearing a nose clip during all trials. The order of conditions was counterbalanced to ensure no order effects were present. They were then instructed to maximally inhale to total lung capacity (TLC), followed by a forced expiration lasting at least 6 seconds and ending when zero flow was reached. This process was performed for each control and resistance condition until 3 trials with FEV_1_ and FVC values were within 150mL for reproducibility, with the best trial for each condition used in the analysis.

During plethysmography testing, participants were seated upright in a sealed box while breathing through a spirometer. Participants were instructed to breathe quietly until achieving a reproducible functional residual capacity (FRC). Volunteers were then instructed to pant at a queued pace (1 Hz) with the spirometer shutter open (R_aw_) and closed (lung volumes) for approximately 5 breaths each. This process was performed for each condition until 3 trials with R_aw_ values were within 10% for reproducibility. R_aw_ was averaged for each experimental condition. Assumptions and calculations for R_aw_ are as follows:

Flow ($\dot{V}$) is defined as

$$\dot{V}=\frac{\left(\text{PATM}-\text{PA}\right)}{\text{R}}$$ (Eq 1)

Where P_ATM_ is atmospheric pressure, P_A_ is alveolar pressure, and R is resistance. Resistance is defined as

$$R=\frac{8\eta \text{l}}{{\pi \text{r}}^{4}}$$ (Eq 2)

where η is the viscosity of the gas, *l* is the length of the airway, and *r* is the radius of the airway. Thus, $\dot{V}$ is more completely defined as

$$\dot{V}=\frac{\Delta P\pi \text{r}4}{8\eta l}$$ (Eq 3)

where ΔP is the pressure gradient. Finally, R_aw_ can be described and measured through body plethysmography as

$$R_{aw}=\frac{\left(PATM-PA\right)}{\dot{V}}$$ (Eq 4)

## Rectangular Area Ratio

Rectangular Area Ratio (RAR) is a geometric analysis that allows the characterization of the shape of the expiratory limb of the flow-volume envelope [20–22]. To measure RAR, a rectangle is drawn bounded by the peak expiratory flow and zero flow at residual volume. A ratio is taken of the area under the expiratory segment of the flow-volume loop to the total area of the rectangle. When the ratio value is greater than 0.5, this indicates the shape of the limb is convex. When the value is below 0.5, this indicates the shape is concave. Each RAR value was obtained using custom-designed software using MATLAB (MathWorks, Natick, MA) to identify the PEF, zero flow, and the area under the expiratory limb of the flow-volume relationship. RAR was calculated as follows [20]:

$$\text{RAR}=\frac{\int_{V@{\dot{V}}_{\text{max}}}^{V@{\dot{V}}_{\text{EE}}}\dot{V}dV-({\dot{V}}_{\text{EE}}\times V_{\text{T}})}{V_{\text{T}}({\dot{V}}_{\text{max}}-{\dot{V}}_{\text{EE}})}$$ (Eq 5)

where $V@{\dot{V}}_{EE}$ and $V@{\dot{V}}_{max}$ are volumes at end-expiratory flow and peak expiratory flow, and *V*_*T*_ is tidal volume.

### Statistical analysis

Differences in pulmonary function variables were compared using a one-way repeated measures ANOVA. All spirometry variables and R_aw_ were examined for correlation with RAR using the Pearson Product-Moment Correlation method. In the case of a significant omnibus test, a Bonferroni post hoc test was used for follow-up analysis (Prism, GraphPad, San Diego, CA).

## Results

**Fig 1:**
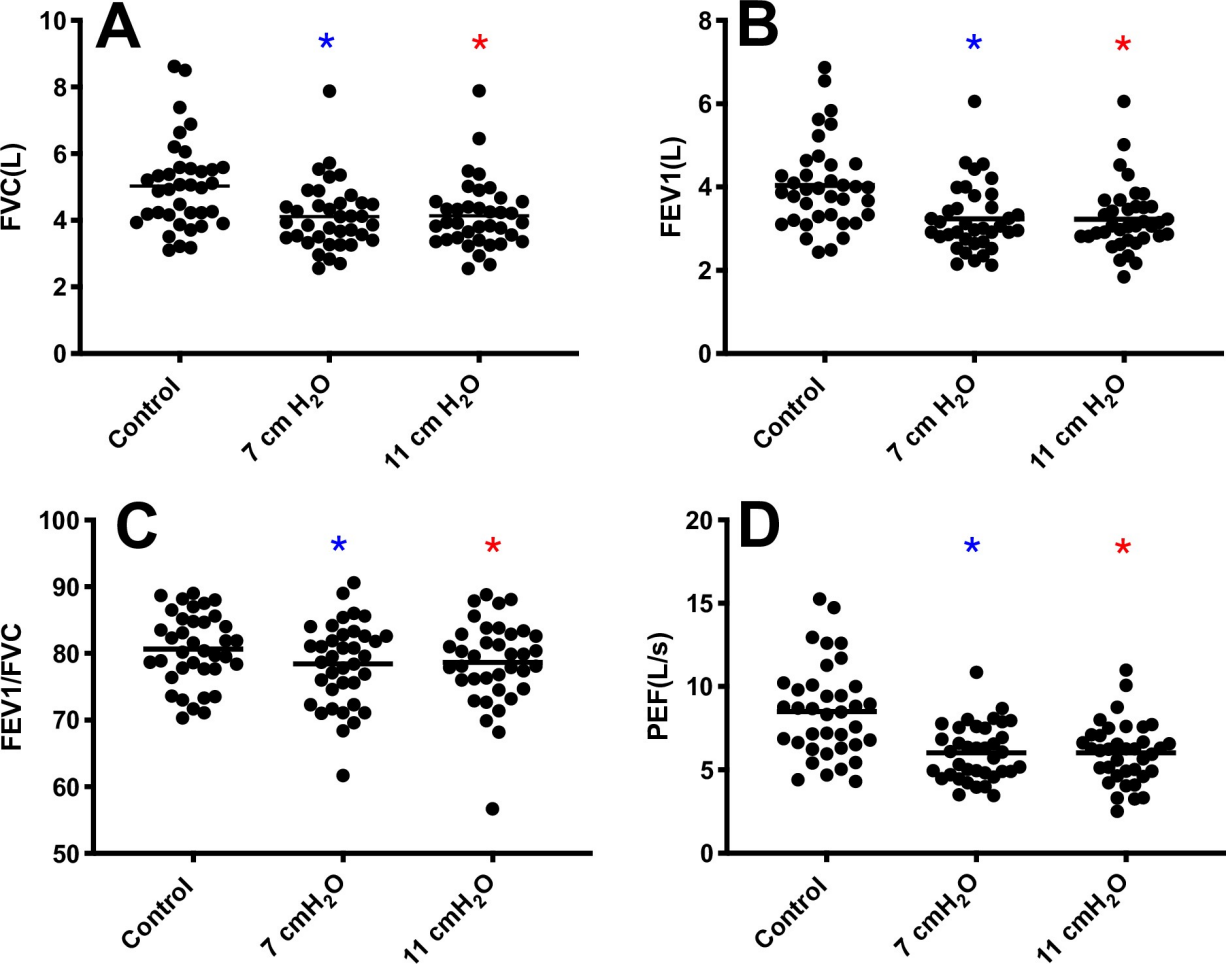
Spirometry variables with and without expiratory loading. **Panel A:** Forced vital capacity (FVC) was lower with 7 and 11 cmH_2_O of loading vs control (F[1.495, 55.31] = 77.71, *p*<0.05). **Panel B:** Forced expired volume in 1 s (FEV1) was lower with 7 and 11 cmH_2_O of loading vs control (F[1.632, 60.38] = 67.54, *p*<0.05). **Panel C:** FEV1/FVC was lower with 7 and 11 cmH_2_O of loading vs control (F[1.870, 69.17] = 5.553, *p*<0.05). **Panel D:** Peak expiratory flow (PEF) was lower with 7 and 11 cmH_2_O of loading vs control (F[1.513, 55.99] = 72.69, *p*<0.05). *Post hoc test showing different from control (*p*<0.05).

Six participants with an FEV_1_/FVC < 0.70 were excluded from analysis on the basis of abnormal spirometry, and this included four from the smaller subset. We examined four primary spirometry variables of FVC, FEV_1_, FEV_1_/FVC, and PEF (n = 37). FVC (F[1.5, 53.4] = 74.5, *p*<0.0001) was reduced at 7 and 11 cmH_2_O vs control (4.11±1.01 and 4.14±1.03 L, respectively vs. 5.03±1.34 L, **Fig 1A**). FEV_1_ was also reduced (F[1.6, 57.5] = 75.3, *p*<0.0001) with 7 and 11 cmH_2_O of loading vs control (3.24±0.81 and 3.23±0.80 L, respectively vs. 4.04±1.05 L, **Fig 1B**). FEV_1_/FVC (F[1.90, 69.9] = 10.6, *p*<0.0068) was reduced with 7 and 11 cmH_2_O of loading vs control (78.5±6.22 and 78.7±6.32%, respectively vs. 80.7±5.35%, **Fig 1C**). PEF (F[1.5, 54.9] = 80.0, *p*<0.0001) was reduced with 7 and 11 cmH_2_O of loading vs control (6.03±1.67 and 6.02±1.84 L/s, respectively vs. 8.50±2.81 L/s, **Fig 1D**). No differences were present between 7 and 11 cmH_2_O for any of the spirometry variables.

**Fig 2:**
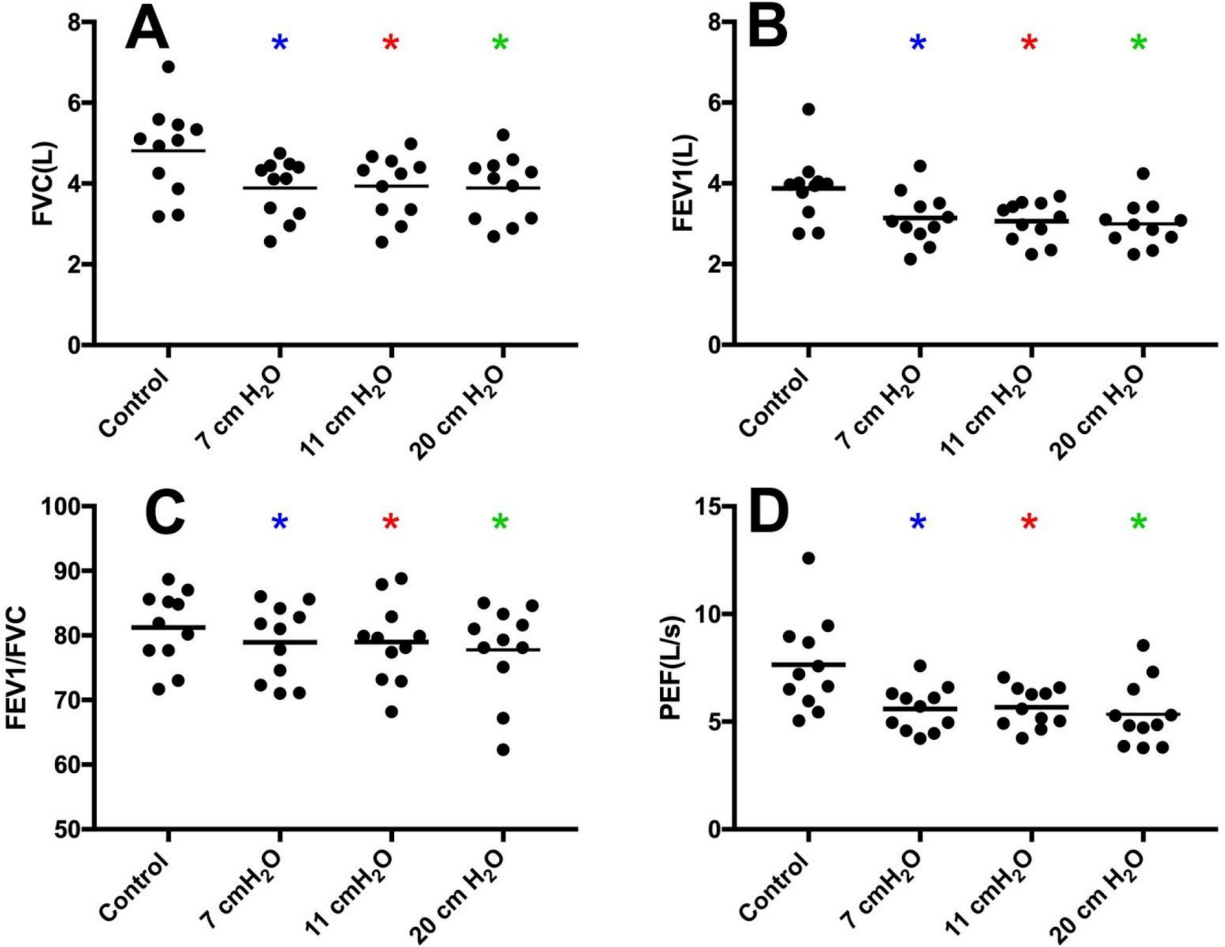
Spirometry data for volunteers with additional imposed expiratory loading of 20 cmH_2_O (n = 11). **Panel A:** FVC was reduced with expiratory resistance (F[1.4, 14.4] = 23.6, *p*<0.05). **Panel B:** FEV_1_ was reduced with expiratory resistance (F[2.0, 20.4] = 17.6, *p*<0.05). **Panel C:** FEV_1_/FVC was reduced with expiratory resistance (F[2.3, 22.6] = 3.9, *p*<0.05). **Panel D:** PEF was reduced with expiratory resistance (F[1.3, 13.2] = 22.0, *p*<0.05). *Different from control (*p*<0.05).

Volunteers with additional imposed expiratory loading of 20 cmH_2_O (n = 11) had reduced FVC (F[1.4, 14.4] = 23.6, *p* < 0.05) at 7, 11, and 20 cmH_2_O vs control (3.89 ± 0.72, 3.94 ± 0.78, and 3.89 ± 0.81 L, respectively, vs. 4.81 ± 1.10 L, **Fig 2A**). FEV_1_ was reduced (F[2.0, 20.4] = 17.6, *p* < 0.05) at 7, 11, and 20 cmH_2_O vs. control (3.14 ± 0.65, 3.07 ± 0.50, and 3.0 ± 0.56 L, respectively, vs. 4.09 ± 0.91 L, **Fig 2B**). FEV_1_/FVC was reduced (F[2.3, 22.6] = 3.9, *p* < 0.05) at 7, 11, and 20 cmH_2_O vs control (78.93 ± 5.81% and 77.78 ± 7.18% vs. 81.23 ± 5.67%, *p* < 0.05, **Fig 2C**). PEF was reduced (F[1.3, 13.2] = 22.0, *p* < 0.05) with 7, 11, and 20 cmH_2_O vs control (5.60 ± 1.05 L/s, 5.67 ± 0.93 L/s, and 5.35 ± 1.53 L/s, respectively, vs. 7.64 ± 2.18 L/s, **Fig 2D**). No differences were present between 7, 11 and 20 cmH_2_O for any of the spirometry variables.

**Fig 3:**
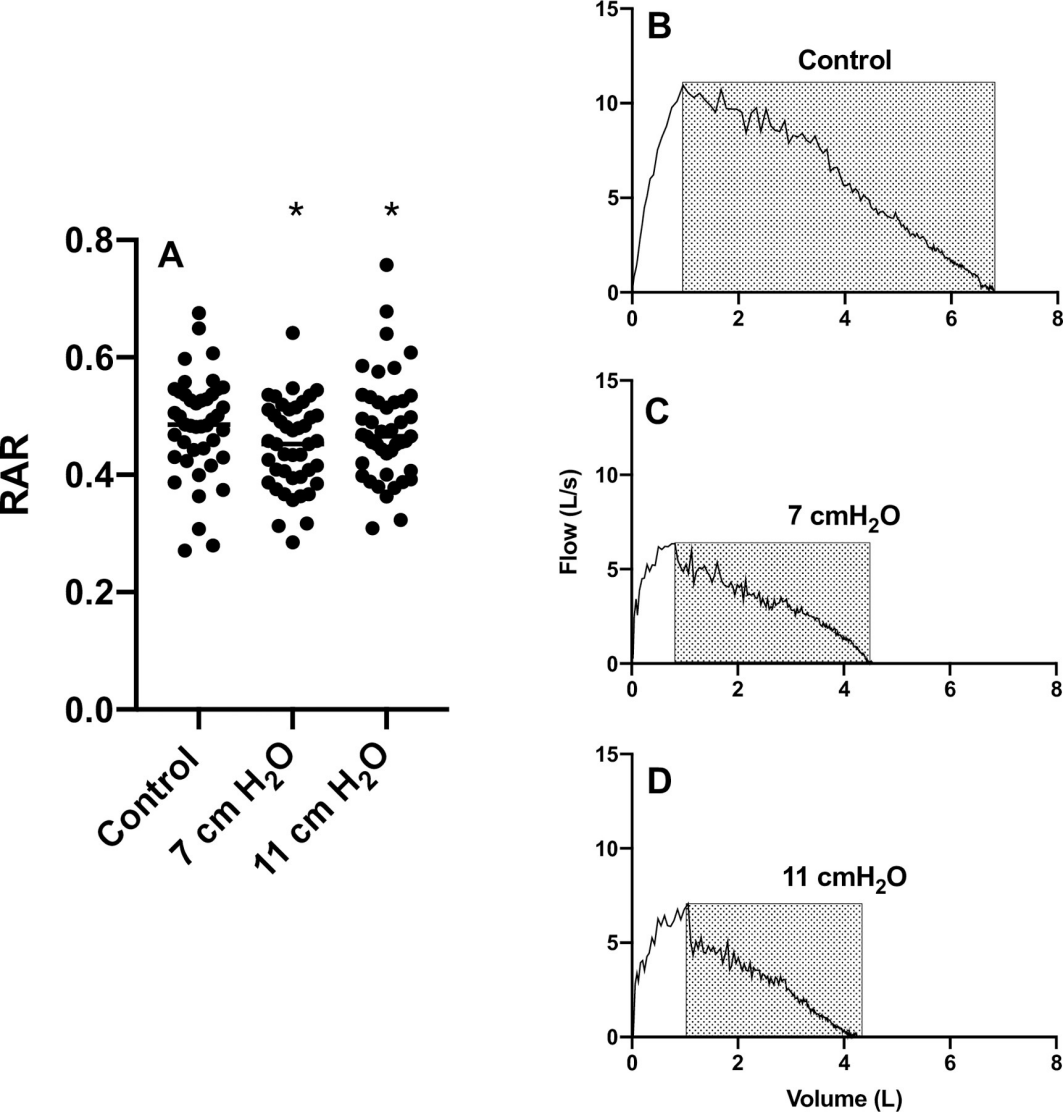
Rectangular area ratio group data and a representative participant. **Panel A** Rectangular Area Ratio (RAR) measured with and without imposed expiratory loading (F[1.855, 77.90] = 3.711, *p*<0.05). **Panel B** A representative participant without any loading in which the RAR = 0.54. **Panel C** The same participant in which the loading of 7 cmH_2_O resulted in RAR = 0.49. **Panel D** The same participant in which the flow-volume relationship was at 11 cmH_2_O loading resulted in RAR = 0.45. *Different from control (p<0.05).

RAR (F[1.855, 77.90] = 3.711, *p* = 0.0319) was reduced at 7 and 11 cmH_2_O vs control (0.45±0.07 and 0.48±0.09, respectively vs. 0.48±0.09, **Fig 3**).

**Fig 4:**
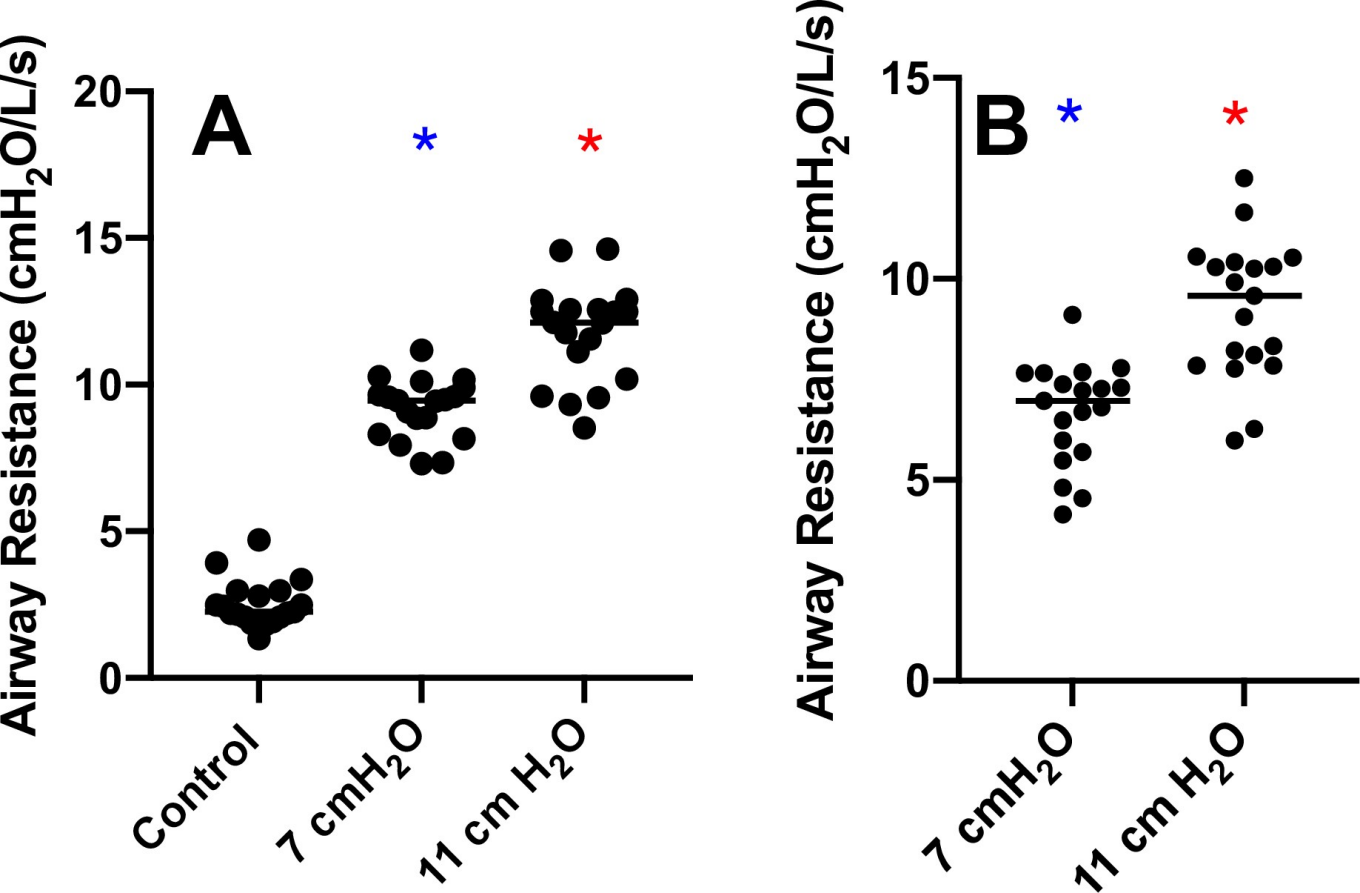
Airway resistance (R_aw_). **Panel A** Airway resistance (R_aw_) measured with and without imposed expiratory loading (F[1.531, 27.55] = 446.0, *p*<0.05). **Panel B** ΔR_aw_ represents R_aw_ with the control values subtracted (t[18] = 10.18, *p*<0.05). *Different from control (p<0.05).

**Fig 5:**
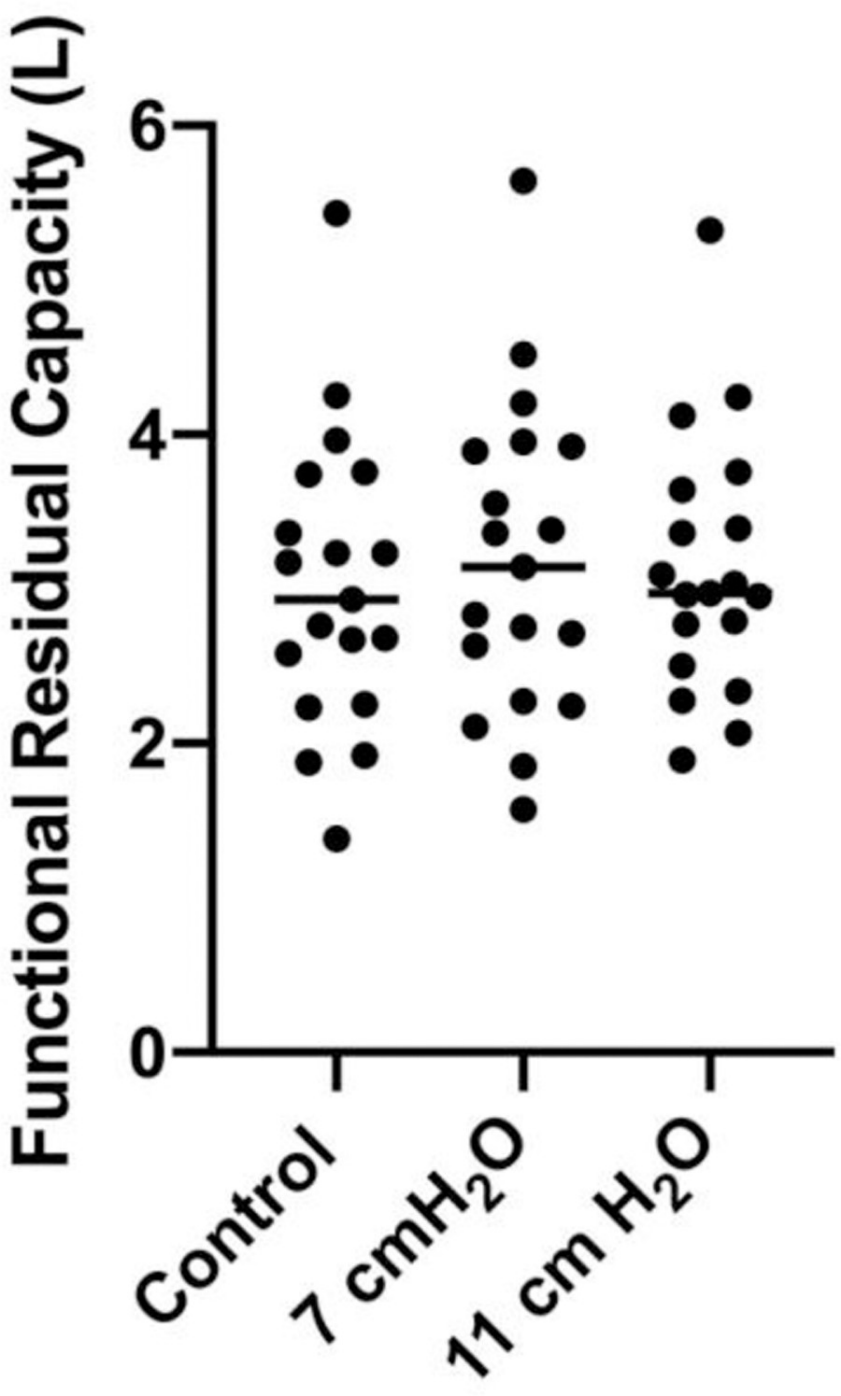
FRC measured with and without imposed expiratory loading.

**Fig 6:**
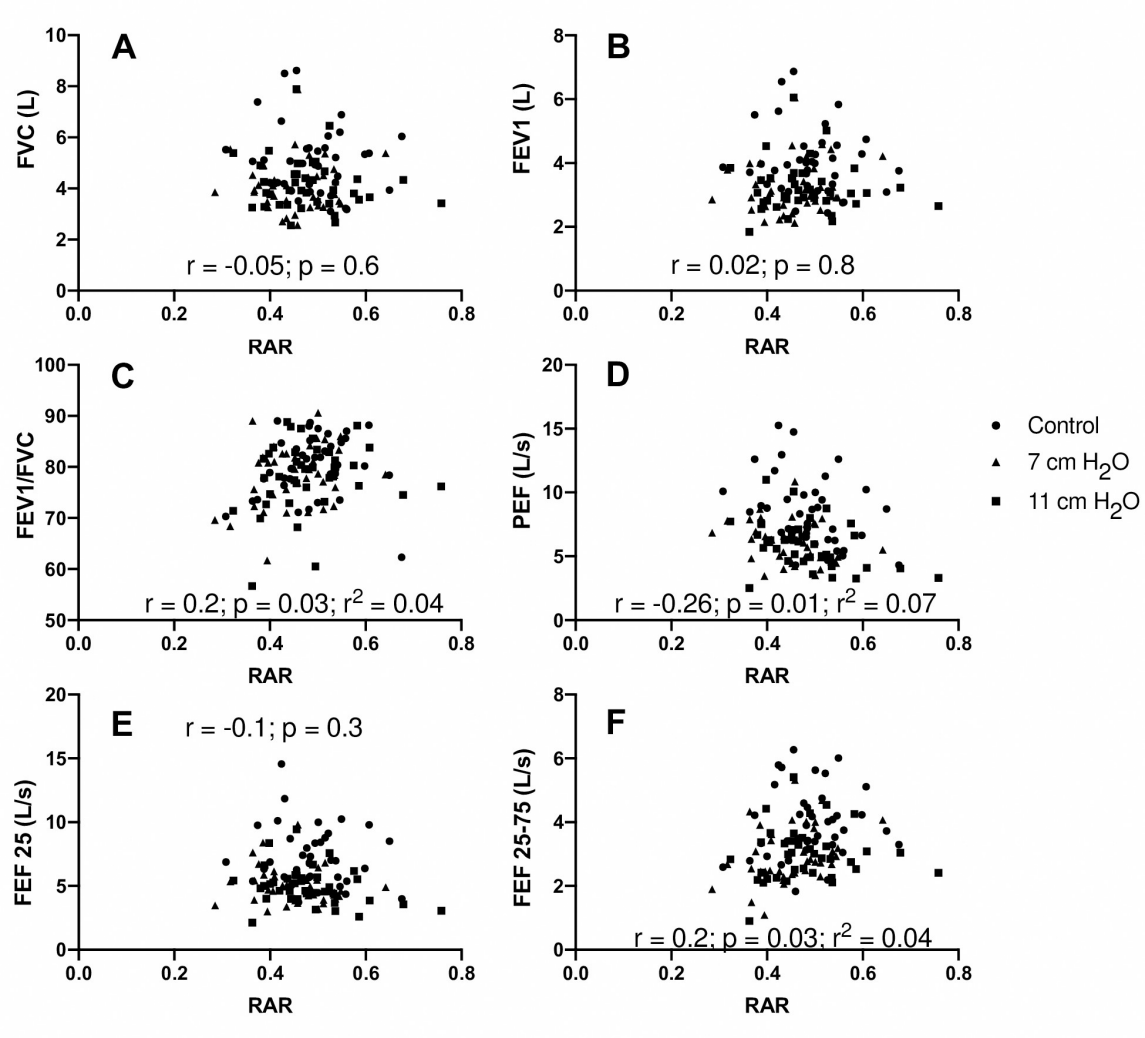
Rectangular Area Ratio (RAR) vs individual primary pulmonary function variables measured with and without imposed expiratory loading. **Panels C, D, F** show a correlation but one that is of no clinical importance. Pearson, p value, and where appropriate the coefficient of determination is included on the figures.

**Fig 7:**
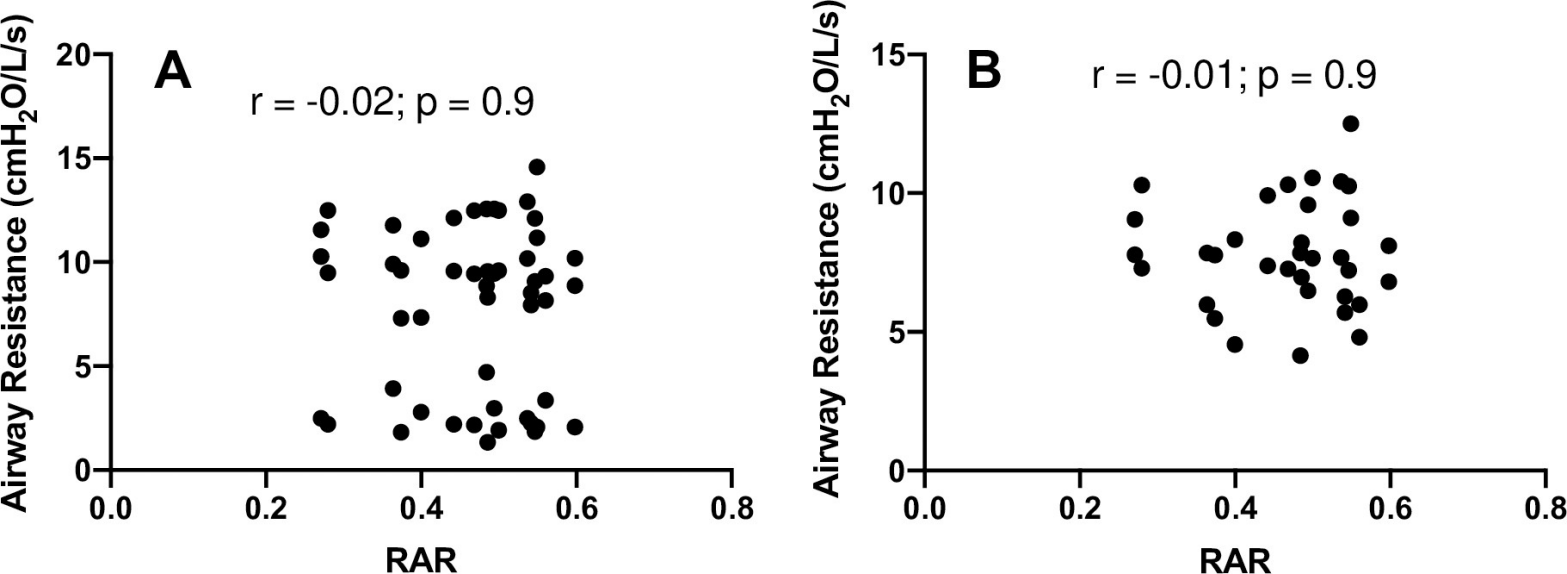
Rectangular Area Ratio (RAR) vs airway resistance variables with and without imposed expiratory resistance. **Panels A** and **B** show no correlation between the variables.

We examined body plethysmography variables including airway resistance (R_aw_) and functional residual capacity (FRC). R_aw_ (F[1.531, 27.55] = 446.0, *p*<0.0001) was increased with 7 and 11 cmH_2_O vs control (9.20±1.02 and 11.76±1.68 cmH_2_O/L/s, respectively vs. 2.53±0.80 cmH_2_O/L/s, **Fig 4A**). Airway resistance for the 7 cmH_2_O (6.67±1.27 cmH_2_O/L/s) and the 11 cmH_2_O (9.23±1.73 cmH_2_O/L/s) conditions were different from one another (t[18] = 10.18, *p*<0.05, **Fig 4B**). FRC (F[1.863, 33.53] = 0.9289, *p* = 0.4) was not different between resistance conditions (**Fig 5**). We measured the relationship between RAR and FEV_1_ (*p =* 0.814 and r^2^<0.0005), FVC (*p =* 0.630 and r^2^ = 0.002), FEV_1_/FVC (*p =* 0.030 and r^2^ = 0.041), PEF (*p =* 0.005 and r^2^ = 0.005), FEF_25_ (*p =* 0.275 and r^2^ = 0.011) and FEF_25-75_ (*p =* 0.027 and r^2^ = 0.043). We also measured the relationship between RAR and R_aw_ (*p =* 0.894 and r^2^<0.0004) and ΔR_aw_ (*p =* 0.974 and r^2^< 0.00001) (**Figs 6 and 7**). None of the variables were related to RAR.

## Discussion

We aimed to measure the effect of imposed expiratory loading on pulmonary function (FVC, FEV_1_, FEV_1_ /FVC, PEF, FRC, and R_aw_) in healthy adults. Imposed expiratory loading resulted in increased R_aw_ and reduced FEV_1_, FVC, and PEF in healthy adults with no clinically significant change in the ratio of FEV_1_ /FVC or FRC. Additionally, RAR did not show a clinically meaningful reduction to suggest alteration in the shape of the flow-volume envelope. There was no perceptible dose-response of imposed loading on pulmonary function, even up to a supra-physiologic loading of 20 cmH_2_O threshold pressure.

### External loading and dynamic airway compression

Imposed resistance at the mouth generates lung function abnormalities that are substantially different to that of patients with resistance within the airways. While expiratory flow limitation results from dynamic airway compression in relatively distal airways in patients with COPD, resistance applied at the mouth can maintain airway pressures, the equal pressure point, and prevent airway compression. Naturally, mitigation of dynamic airway compression is one of the mechanisms by which pursed lip breathing is effective [23, 24]. We suspect this is the mechanism for why the flow-volume loop failed to show a scooping, or concave profile, similar to that commonly found in COPD where FEV_1_/FVC is reduced.

Further evidence to this point is provided by experiments in which negative pressures are imposed at the mouth in patients with COPD. Applying negative expiratory pressures during tidal breathing in people without flow limitation can improve flow as long as the mouth pressures are modest, such as -5 cmH_2_O [25]. In individuals with severe flow limitation, imposed negative expiratory pressure leads to decreases in flow [25] due to exacerbation of dynamic airway compression [2]. At greater unloading (-10 cmH_2_O/L/s), dynamic airway compression is accentuated [2]. This also increases the sensation of dyspnea and provides an alternative method that may be more closely associated with dyspnea scores than spirometry measurements [26].

### Absence of dose-response for external threshold loading

There was no apparent spirometry dose-response caused by imposed threshold expiratory resistance. This is despite a clear dose-response in the airway resistance itself. The majority of the decline in lung function appeared to occur at moderate flow limitation. While surprising, it may be that expiratory loading of 11 and 20 cmH_2_O only serve to raise airway pressures, such that the equal pressure point is maintained similarly to that of when 7 cmH_2_O is imposed on the participant. This again highlights a key limitation in our experiment where resistance outside of the airways deviates substantially from resistance within an airway.

It is not clear what external loading would be necessary to produce a dose-response for spirometry. Again, reduced aperture loading appears to accomplish this for modelling upper airway obstruction [18, 19], however we were unable to reproduce the behavior using a threshold device. Part of the explanation may be that healthy people are capable of very high maximal expiratory pressures (MEP) sufficient to overcome just about any commercial threshold device. In healthy individuals, MEP is upwards of 125 cm H_2_O [27] and even in COPD MEP may be ~75 cmH_2_O [28]. Providing threshold loading with an opening pressure closer to the MEP would certainly further increase the work of breathing and provide more resistance. This amount of resistance placed outside the airways, however, is magnitudes larger than what is present in obstructive disease. There may be no easy solution to impose obstruction outside of the airways.

### Indicators of obstruction and restriction

FEV_1_/FVC was reduced with imposed loading, however only by 3–4%. Furthermore, mean FEV_1_/FVC did not fall below the obstructive threshold. These modest changes are due to the proportional reduction in FEV_1_ and FVC. The reduction in FVC results from mouth pressure at low lung volumes failing to maintain threshold pressure. Interestingly, the FVC did not differ across the conditions, however it may be a signal:noise limitation for such small change in residual volume. Reductions in FVC are also common in restrictive lung diseases, with minimal changes, or even increases in FEV_1_/FVC [29]. In this manner, external loading has some limited application for restrictive disease, however the absence of reduced TLC or increase in lung stiffness makes this largely inappropriate as a model.

### Graphical analysis of the flow-volume relationship

Chronic obstructive diseases are often progressive with no cure. Therefore, lung function can be expected to worsen over time, and it is no surprise that spirometry is used to monitor progression. However, monitoring of symptoms, exercise tolerance, comorbidity, and smoking habits are also highly recommended to guide treatment, and assess management options [30, 31]. While spirometry alone cannot give a complete picture of the progression of COPD, graphical analysis of the flow-volume relationship [20, 32] may provide an important supplement. With this in mind, we examined the relationship of RAR with spirometry variables that are currently used to diagnose and monitor restrictive and obstructive diseases. We found no relationship between RAR and these variables (**Figs 6 and 7**). The implication is that RAR may be providing additional information about the flow-volume envelope not captured by traditional variables. To test this hypothesis, longitudinal data are needed to track progression of lung function. Ideally, this may constitute monitoring healthy people and patients with COPD not just with lung function tests, but other clinical outcomes such as imaging, quality of life, and, of course, tracking hospitalization and mortality.

## Conclusions

We examined the effect of external expiratory loading on airway resistance and the flow-volume relationship. Expiratory loading resulted in a dose-response increase in the total airway resistance and a reduction in FEV_1_ and PEF. With external loading, FVC decreased similarly to FEV_1_ and, therefore, FEV_1_/FVC did not change. There was no dose-response in spirometry variables with 7, 11, or even 20 cmH_2_O threshold loading. Graphical analysis of the expiratory segment of the flow-volume relationship did not reveal a clinically meaningful change in the shape of the envelope. The rectangular area ratio was not related to any pulmonary function variables, thus there was no perceptible concavity, or scooping effect, seen in the expiratory limb of the flow-volume envelope. This may be attributed to the mitigation of dynamic airway compression caused by the additional load maintaining airway patency. The absence of concavity in the flow-volume relationship renders expiratory loading inappropriate to mimic obstructive pulmonary disease. The intervention approach may, however, be a useful strategy to increase work of breathing. Additionally, threshold loading may also be useful to induce dynamic hyperinflation in otherwise healthy volunteers. This has already been demonstrated using varied methods of expiratory loading and provides a model to study abnormal lung mechanics without the systemic complexities in patients with obstructive diseases.

## References

[1] Langer D, Ciavaglia CE, Neder JA, Webb KA, O’Donnell DE. Lung hyperinflation in chronic obstructive pulmonary disease: mechanisms, clinical implications and treatment. Expert review of respiratory medicine. 2014;8(6):731–49. doi: 10.1586/17476348.2014.949676 25159007

[2] O’Donnell DE, Sanii R, Anthonisen NR, Younes M. Effect of dynamic airway compression on breathing pattern and respiratory sensation in severe chronic obstructive pulmonary disease. American Review of Respiratory Disease. 1987;135(4):912–8. doi: 10.1164/arrd.1987.135.4.912 3565938

[3] O’Donnell DE. Hyperinflation, dyspnea, and exercise intolerance in chronic obstructive pulmonary disease. Proceedings of the American Thoracic Society. 2006;3(2):180–4. doi: 10.1513/pats.200508-093DO 16565429

[4] Barreiro E, Gea J. Respiratory and limb muscle dysfunction in COPD. COPD: Journal of Chronic Obstructive Pulmonary Disease. 2015;12(4):413–26. doi: 10.3109/15412555.2014.974737 25438125

[5] Makarevich AE, Lemiasheuskaya SS, Poctavcev AJ, Lemeschewskij AI, Nedvedz M. The dynamics of respiratory muscle changes during the progression of chronic obstructive pulmonary disease. Adv Clin Exp Med. 2014;23(3):381–94. doi: 10.17219/acem/37129 24979509

[6] Maltais F, Decramer M, Casaburi R, Barreiro E, Burelle Y, Debigare R, et al. An official American Thoracic Society/European Respiratory Society statement: update on limb muscle dysfunction in chronic obstructive pulmonary disease. American journal of respiratory and critical care medicine. 2014;189(9):e15–e62. doi: 10.1164/rccm.201402-0373ST 24787074 PMC4098112

[7] O’Donnell DE, Laveneziana P, Webb K, Neder JA. Chronic obstructive pulmonary disease: clinical integrative physiology. Clinics in chest medicine. 2014;35(1):51–69. doi: 10.1016/j.ccm.2013.09.008 24507837

[8] Cooper CB. Airflow obstruction and exercise. Respiratory medicine. 2009;103(3):325–34. doi: 10.1016/j.rmed.2008.10.026 19071004

[9] Katajisto M, Kupiainen H, Rantanen P, Lindqvist A, Kilpeläinen M, Tikkanen H, et al. Physical inactivity in COPD and increased patient perception of dyspnea. International journal of chronic obstructive pulmonary disease. 2012;7:743. doi: 10.2147/COPD.S35497 23152679 PMC3496537

[10] Nussbaumer-Ochsner Y, Rabe KF. Systemic manifestations of COPD. Chest. 2011;139(1):165–73. doi: 10.1378/chest.10-1252 21208876

[11] Eberlein M, Schmidt GA, Brower RG. Chest Wall Strapping. An Old Physiology Experiment with New Relevance to Small Airways Diseases. Annals of the American Thoracic Society. 2014;11(8):1258–66. doi: 10.1513/AnnalsATS.201312-465OI 25172621 PMC5469355

[12] Spahija J, Grassino A. Effects of pursed-lips breathing and expiratory resistive loading in healthy subjects. Journal of Applied Physiology. 1996;80(5):1772–84. doi: 10.1152/jappl.1996.80.5.1772 8727566

[13] Aliverti A, Iandelli I, Duranti R, Cala SJ, Kayser B, Kelly S, et al. Respiratory muscle dynamics and control during exercise with externally imposed expiratory flow limitation. Journal of Applied Physiology. 2002 May 1;92(5):1953–63. doi: 10.1152/japplphysiol.01222.2000 11960945

[14] Aliverti A, Kayser B, Macklem PT. A human model of the pathophysiology of chronic obstructive pulmonary disease. Respirology. 2007 Jul;12(4):478–85. doi: 10.1111/j.1440-1843.2007.01106.x 17587412

[15] Bretonneau Q, Pichon A, de Bisschop C. Effect of expiratory loaded breathing during moderate exercise on intercostal muscle oxygenation. Multidisciplinary Respiratory Medicine. 2020 Jan 28;15(1). doi: 10.4081/mrm.2020.702 33154819 PMC7610065

[16] Urban MH, Mayr AK, Schmidt I, Margulies E, Grasmuk-Siegl E, Burghuber OC, et al. Induction of dynamic hyperinflation by expiratory resistance breathing in healthy subjects–an efficacy and safety study. Experimental Physiology. 2021 Feb;106(2):532–43. doi: 10.1113/EP088439 33174314 PMC7894562

[17] Iandelli I, Aliverti A, Kayser B, Dellacà R, Cala SJ, Duranti R, et al. Determinants of exercise performance in normal men with externally imposed expiratory flow limitation. Journal of Applied Physiology. 2002 May 1;92(5):1943–52. doi: 10.1152/japplphysiol.00393.2000 11960944

[18] Melissant C, Lammers J, Demedts M. Relationship between external resistances, lung function changes and maximal exercise capacity. European Respiratory Journal. 1998;11(6):1369–75. doi: 10.1183/09031936.98.11061369 9657581

[19] Wassermann K, Gitt A, Weyde J, Eckel HE. Lung function changes and exercise-induced ventilatory responses to external resistive loads in normal subjects. Respiration. 1995;62(4):177–84. doi: 10.1159/000196444 8578012

[20] Ma S, Hecht A, Varga J, Rambod M, Morford S, Goto S, et al. Breath-by-breath quantification of progressive airflow limitation during exercise in COPD: a new method. Respiratory medicine. 2010;104(3):389–96. doi: 10.1016/j.rmed.2009.10.014 19931441

[21] Porszasz J, Carraro N, Cao R, Gore A, Ma S, Jiang T, et al. Effect of tiotropium on spontaneous expiratory flow–volume curves during exercise in GOLD 1–2 COPD. Respiratory physiology & neurobiology. 2018 May 1;251:8–15. doi: 10.1016/j.resp.2018.02.006 29438808

[22] Varga J, Casaburi R, Ma S, Hecht A, Hsia D, Somfay A, et al. Relation of concavity in the expiratory flow-volume loop to dynamic hyperinflation during exercise in COPD. Respiratory physiology & neurobiology. 2016 Dec 1;234:79–84. doi: 10.1016/j.resp.2016.08.005 27575552

[23] Mueller RE, Petty TL, Filley GF. Ventilation and arterial blood gas changes induced by pursed lips breathing. Journal of applied physiology. 1970;28(6):784 doi: 10.1152/jappl.1970.28.6.784 5419502

[24] Thoman RL, Stoker GL, Ross JC. The efficacy of pursed-lips breathing in patients with chronic obstructive pulmonary disease. American Review of Respiratory Disease. 1966;93(1):100–6. doi: 10.1164/arrd.1966.93.1.100 5901381

[25] Koulouris N, Valta P, Lavoie A, Corbeil C, Chassé M, Braidy J, et al. A simple method to detect expiratory flow limitation during spontaneous breathing. European Respiratory Journal. 1995;8(2):306–13. doi: 10.1183/09031936.95.08020306 7758567

[26] Eltayara L, Becklake MR, Volta CA, Milic-Emili J. Relationship between chronic dyspnea and expiratory flow limitation in patients with chronic obstructive pulmonary disease. American journal of respiratory and critical care medicine. 1996;154(6):1726–34. doi: 10.1164/ajrccm.154.6.8970362 8970362

[27] Black LF, Hyatt RE. Maximal respiratory pressures: normal values and relationship to age and sex. American review of respiratory disease. 1969;99(5):696–702. doi: 10.1164/arrd.1969.99.5.696 5772056

[28] Terzano C, Ceccarelli D, Conti V, Graziani E, Ricci A, Petroianni A. Maximal respiratory static pressures in patients with different stages of COPD severity. Respiratory research. 2008;9(1):8. doi: 10.1186/1465-9921-9-8 18208602 PMC2244619

[29] Ranu H, Wilde M, Madden B. Pulmonary function tests. The Ulster medical journal. 2011;80(2):84. 22347750 PMC3229853

[30] Bellamy D, Bouchard J, Henrichsen S, Johansson G, Langhammer A, Reid J, et al. International Primary Care Respiratory Group (IPCRG) Guidelines: Management of Chronic Obstructive Pulmonary Disease (COPD). Primary Care Respiratory Journal. 2006;15(1):48–57. doi: 10.1016/j.pcrj.2005.11.003 16701758 PMC6730681

[31] Van Den Bemt L, Schermer T, Smeele I, Bischoff E, Jacobs A, Grol R, et al. Monitoring of patients with COPD: A review of current guidelines’ recommendations. Respiratory Medicine. 2008;102(5):633–41. doi: 10.1016/j.rmed.2007.12.014 18242067

[32] Lee J, Lee C-T, Lee JH, Cho Y-J, Park JS, Oh Y-M, et al. Graphic analysis of flow-volume curves: a pilot study. BMC pulmonary medicine. 2016;16(1):18. 26801632 10.1186/s12890-016-0182-8 PMC4724104

